# Analysis of Wellbeing in Nongovernmental Organizations’ Workplace in a Developed Area Context

**DOI:** 10.3390/ijerph17165818

**Published:** 2020-08-11

**Authors:** Virginia Navajas-Romero, Lorena Caridad y López del Río, Nuria Ceular-Villamandos

**Affiliations:** Department of Statistics and Operations Research, Business Organization and Applied Economics, Universidad de Córdoba, 14014 Córdob, Spain; d92calol@uco.es (L.C.yL.d.R.); nuria.ceular@uco.es (N.C.-V.)

**Keywords:** job demands, job control, social support, wellbeing, NGOs

## Abstract

An extremely useful theoretical approach to understanding the nature of work, health, and wellbeing is the job demand–control (JDC) model and the job demand–control–support (JDCS) model. In order for professional workers in the nongovernmental organization (NGO) sector to do their job, it is necessary for them to have a feeling of wellbeing. Despite this, in Europe, studies regarding the effects of the JDCS model in relation to workers’ wellbeing have not been carried out. This study is expected to fill this important gap in research by analyzing the relationship of wellbeing with work demands, work control, and social support. In order to corroborate the proposed hypotheses, an analysis of these constructs in employees in European nongovernmental organizations (NGOs) was developed and, using structural equation models, these relationships were tested. The results confirm the main hypothesis of the job demand–control–support (JDCS) model and the causal relationship among physical and psychological demands, work control, and support from supervisors and colleagues with the level of employee wellbeing.

## 1. Introduction

Due to the global changes in families and communities within society, nongovernment institutions have become one of the basic pillars for public wellbeing. The tasks they perform are the origin of many basic social functions. However, their purpose is not to replace public administrations. The aims of nongovernmental institutions are diverse. Although they remain within general public wellbeing, they have key roles such as humanitarian functions, the defense of human rights, the management of economic development, actions during natural disasters, and protection of the environment [1]. This situation has led to the rapid growth of NGOs in the international landscape in the last three decades. In line with this idea, the Union of International Associations estimates that there are almost forty thousand active international NGOs in 2020, and more than a thousand new international NGOs are added every year in the United States of America alone [2]. Additionally, the World Bank shows that in the last twenty years, projects financed that involve NGOs or other civil organizations have increased from 21% to 90%, and, additionally, more than 20% of all bilateral aid is managed through NGOs [3].

The importance of growth and the relevance of the sector to society are fundamental, and maintaining the optimal conditions of NGO workers is a priority in this sector [4]. At an internal organizational level, there is a lot of competition within the sector itself for resources and to maintain an impeccable image [5]. In addition to this, there is the need to work with limited resources, as the financial support that NGOs receive from citizens is indispensable and essential for the completion of projects. As such, the management of resources influences the level of satisfaction that is attained later on due to their work in civil society and the state [6,7]. In addition to this, the workers of the NGOs provide their services by means of direct interaction with the community [7,8].

A basic element for humans is the wellbeing of everyone in their own work activities. In the study of job wellbeing, it is necessary to analyze basic aspects derived from each job, such as the income received, the demands associated to their workplace position, the pressure in meeting deadlines, the difficulties in the tasks assigned, the skills required, and the workplace atmosphere, which are among the most important factors. The relationship between wellbeing in the workplace and the employee’s personal wellbeing is complex, mainly due to work conditions affecting the person’s quality of life [9]; also, they may affect aspects such as health and stress [10].

From a theoretical point-of-view, wellbeing conceptualizes the assessment that people perceive of their own quality of life [11], including their workplace situation, as well as the personal conditions, in accordance with an individual and personal organization of values, goals, and aspirations [12,13,14,15]. From this perspective, academic literature should focus on the analysis of the individuals’ work-life, and this should be a basic concern for society [16]. The job demand–control (JDC) model identifies two working condition variables: the demands upon employees and the control of the activities [17]. In the eighties, a third dimension was added to the model: the social aspects [18,19]; this led to the job demand–control–support (JDCS) model, also known as the “job-strain model”. These theoretical structures identify the key dimensions of labor activity, that is, the organizational or psychological demands (job demands), the independence that employees enjoy regarding their functions, the tasks and skills required for this position, and, finally, the social support from the worker’s supervisor and colleagues.

The great advantage of these models is summarized in the approach that allows the analysis of the job characteristics related to workplace health in a holistic way [20]. In accordance with academic literature, employees of NGOs present low job demands, especially when this is followed by a high-control job that has low personal independence and high social support; this is known as the iso-strain hypothesis [21]. In a conventional model of human resources management, it is a “relaxed” job (i.e., low job demands and low job control) that does not provide employees with such intrinsic motivation, so they experimented with a high level of burnout, together with a decline in productivity. This induces a low work-engagement and desire to move to another job [22]. Following this idea, most of the academic literature link NGOs to low productivity and an unclear decision process [23,24,25].

Notwithstanding, despite the undoubted usefulness of the model, in order to analyze the interactive results of job demands and controls and social support, more empirical evidence is required for certain specific occupations, especially in the case of those with a significant contribution to society. The maintenance of NGO workers’ wellbeing is important in order to improve what these institutions contribute to society. With this approach, it should be possible to identify deviations between working conditions and wellbeing, and how the organizational structures are influenced by this state of affairs. This should produce theoretical and practical implications in human resource management within the NGOs, contributing to the discovery of which organizational models, compared to the usual firm’s approaches, lead to higher levels of wellbeing. The general objective is, thus, to link the interrelations between the labor demand, the job control, and the social support to this segment of the workforce, and to explore if these models diverge, in their final results, from the conventional models, taking into account the job environment, as stated in the literature review.

Undoubtedly, employees in NGOs perform activities that are particularly interesting for society, due to their importance for the overall development of the welfare state [26]. Thus, the activities of NGOs are linked to low job demands, low job control, and high social support. The job demands of this sector are highlighted by having direct contact with people, teamwork, and collaboration with the social environment [27,28]. In addition, these institutions are prone to develop high job control [29]. Following these ideas, clear government supervision, due to strict operational and supranational control, continues to be enhanced in the NGO sector by its internal organization, which should be based on transparency and public support, beneath numerous regulations and protocols. This state of affairs restricts the independence of these professional workers [29]. Finally, another pillar of the theoretical model is based on social support, as it is society that shows approval for many of the NGOs’ initiatives by means of financial funds allocated to these institutions [30]. In this context, social support is very important because, in the case of catastrophes, obtaining financial resources allows for an immediate response on the entity’s behalf [31]. From the internal aspect of the business, various studies indicate that relying on the support of superiors and/or colleagues contributes decisively to improve job wellbeing [32,33].

The NGO sector should aim to reach an efficient and sustainable human resource management model. To attain this general goal, which can be considered a strategic paradigm, the management model has to lead, as near as possible, to the optimal point of wellbeing in the workplace as a consequence of its beneficial influences associated with the tasks performed globally [34]. Job wellbeing is a wide-reaching concept that, in recent decades, has been analyzed in [35]. However, by being a complex concept, it creates difficulties, mainly due to the lack of definition and tools for measurement [36]. To solve these difficulties, the analysis should be considered with a multidisciplinary focus. This research adopts a eudaemonic perspective of psychological wellbeing [37] linked to three perspective factors: enthusiasm, vitality or strength, and, finally, pleasure or serenity. The composition of wellbeing is found in the model presented in [38], which was used by the World Health Organization (WHO; 1998) to prepare a wellbeing index. Wellbeing is one of the most researched issues by academics from the JDCS model context.

If the strategic model in the human resource management of NGOs is not adequate, the consequences the worker may suffer due to the described panorama are the following: stress, alienation, low organizational commitment, lack of internal motivation, and burnout, as the most important. Studies carried out on other occupations propose that the connection of heightened job demands in the presence of low independence can provoke a reduction of wellbeing. The workers, as well as the organizations, can have advantages derived from the improvement in wellbeing. Specifically, organizations that have employees with low wellbeing are subject to negative effects because these employees tend to underperform, and, thus, are less productive. Additionally, the decision process is biased, without a commitment to quality, and they are more inclined to absenteeism [39].

Unlike other sectors, NGO employees offer an immaterial service to society that is indissolubly related to the professional who performs it. A high level of wellbeing originates positive effects, both for the employee and for the organization [40]. However, it is important not to forget that the activities these institutions perform may have uneven results due to the different cultural, economic, or social contexts in which they take place [41]. The divergence among the results obtained while working and those expected by the worker, the organization, or the government may affect the worker’s wellbeing in internal aspects such as motivation and challenge [42]. Here, the usefulness of this study can be found in the idiosyncrasy of the profession and in the added value that the activities contribute to society as a whole. Most research performed regarding NGOs is based on the quantification of the impact of the service quality in society, focusing mainly on obtaining economic results, development, or satisfaction [43,44]. Another branch of literature deals with the study of financing and the relationship with self-protection that employees who control the internal information of the organization perform in their work, ensuring that no organizational failures occur or that no poor image is projected [45]. According to [46], the analysis of the service quality compares the performance of the employees with the satisfaction induced in their customers or society. However, in the European context, there is a small amount of empirical research that analyzes the particular effects of the JDCS model as a multiplicative model related to wellbeing in this sector. Academic literature has not fully developed the interactions between the human resource management of these institutions with the status of their own workforce [47], and it is not frequent to find publications related to the JDCS model. In this work, there is a summary of the state-of-the-art on the management of different human resource models used in European NGOs, and the influence on the workers’ wellbeing. NGO management is usually aware of the sustainability of the environment, enhancing social values and cultural diversity, and, of course, public ethics, and these topics are more frequently found in academic papers. Following this line of thought, the analysis of NGOs can provide new ideas about the general management of human resources, which would be alien to for-profit companies. Additionally, they challenge some well-established techniques used in running institutions. This denotes that the consequences of aspects related to job demands, job control, and social support need to be further studied in activities with a relevant impact on society. Consequently, the academic literature about human resource management in NGOs is scarce, and it is mostly devoted to the efficiency of their internal organization and procedures. In order to cover this important research gap, this paper aims to contribute to these workplace wellbeing studies by analyzing the situation of NGO employees in Europe, taking into account their perceptions and those of the established organizations. Additionally, we study the human resource organization model of NGOs in Europe and, possibly, in developed countries, taking into account the effects of job demands on wellbeing, using a large sample of NGO employees in Europe, as well as looking at the moderating role that different internal aspects play (skill discretion and authority decision) and the importance of external factors (social support of coworkers and supervisors).

## 2. Theoretical Framework

### 2.1. JDCS Model

This approach analyzes the wellbeing situation of NGO employees in Europe using the theoretical framework established by the job demand–control–support (JDCS) model proposed by [17,18,48]. Different researchers have used this model to explain the effects of labor demands on workers’ wellbeing [49,50]. The specification of the JDCS model proposes a causal relation between these demands and wellbeing, although these effects can be enhanced or diminished depending on the degree of control that the employee can exert on the job conditions and on the social support enjoyed. JDC and JDCS models identify the key dimensions of labor activity; that is, job demands can induce physical and psychological stress linked to new and nonforecasted activities that appear as necessary for the organization. Another important aspect is the worker’s control over his daily activities; this is related to individual autonomy over the organization of tasks and how he/she can plan and develop them every day. This control over their jobs is linked to the employee’s authority in the decision-making process; it refers to the possibility of deciding the important aspects of their obligations, the control over their own initiatives, and how to employ their personal abilities. Demands in the workplace and the presence or lack of autonomy are included in Karasek’s (1979) original model, with the tension aspects of labor relations [17]. Consequently, in terms of labor demands and their control, four situations related to the psychological wellbeing can be presented: (1) Workplaces with high labor demands that enjoy a large margin in proposing and implementing their own proposals originate an increase in creativity, better decisions, and an increase in labor participation and general wellbeing. Many professional jobs participate in these properties of independence and autonomy, and they are frequently found in NGO activities. (2) Jobs that are characterized by low workplace demands and high control of their activities originate a higher degree of wellbeing, although they can reduce overall productivity and efficiency. (3) Situations with high labor demands and low control are linked to interpersonal tensions that could originate negative effects on the workers’ wellbeing and even their health. Neffa (2015) relates this kind of jobs to machinery operators, office clerks, hotel staff, and, in general, manual and repetitive tasks [51]. (4) Placements with low job demands and control upon their activities originate a clear loss of motivation, routine habits, and diminished abilities on the workers [52]. This state of affairs originates from a poor job environment and is linked to staff with low qualifications, such as cleaners, porters, and guards. Absenteeism and rotation in the workplace are associated with these situations.

### 2.2. Job Demands, Control and Social Support NGO Workers

According to physical job demands, the activities of NGO workers involve actions with low job demands. The tasks are heterogeneous, including economic development, project management, environment protection, and humanitarian aid in conflict zones. In these situations, the NGOs may place workers’ lives at risk [53]. From a psychological perspective, Hamdan, Talla, Shobaki, and Abu-Naser (2020) presented a paper about international NGOs, where to achieve success, workers should have a clear and broad picture of the whole organization and the speed and timetables of the activities to be carried out to be able to jump onto the opportunities that arise in every-day developments [54]. The pressure exercised by society itself and the supranational bodies induce consequences such as excessive workloads and the lack of resources, affecting worker productivity, mainly caused by the pressure of time and deadlines, which results in low wellbeing [55]. However, job demands are not directly related to the results; in fact, they do not only depend on the worker’s job performance, but also aspects such as the democratic level, economic wealth or resources of the recipient country, and the geographical distance from the donor country, which produces lower wellbeing levels [56]. In general, in the long-term, this causes a drop in motivation and the rate of return to society. NGO attention services are needed around the clock and every day. In this sense, a large personal commitment is demanded from the workers [57,58]. This unstable situation causes a loss of motivation and productivity, as well as a high degree of burnout, low work-engagement, and desire to move to another job [22]. However, there is an increase in the number of experts who seek to work in the nonprofit economy, mainly for altruistic reasons, but also as professional milestones [59], and, especially, to participate in international corporate volunteering programs [60]. In NGOs, an increased turnaround and temporality in staff is a nondesirable situation, as pointed by Chacón and Dávila (2001) [61]. This state of affairs has induced NGOs to define new strategies to hire and retain talent [62].

Another characteristic of NGOs is their centralized organization, as stated by several authors such as Brown et al. (2012) [63] and Lindenberg and Bryant (2001) [64]. This is a fact today, where some institutions are structured in several regional federations or networks, with their own centralized organization. For example, Nature Conservancy is a totally centralized institution, while Oxfam is a loose confederation of many independent organizations, coordinated by an international secretariat. CIVICUS is another federation of autonomous institutions with several shared values [63,65]. This variability in NGOs is associated with their heterogeneous set of objectives, and to their internal assessment methods [66]. In order to achieve those objectives, due to the large number of skills required, professional workers generally have little independence when carrying out their work. These organizations have resorted to “learning in the workplace” as a means of developing and attracting human capital [67]. Besides, it is well-known that it is difficult to work with governments because they lessen the NGOs’ independence [68]. NGO employees have to deal with tensions to comply with regulations of different kinds, together with their own aims and objectives [69]. Although these organizations offer chances to influence governments, they greatly restrict their ability to make decisions [70]. Low job control and the complex social system the sector finds itself in can unleash a deterioration of the job environment [62], which leads to absenteeism, depression, turnover, burnout, absenteeism, and low job turnout and productivity [71]. This scenario impacts the quality of service that NGOs offer [72], leading NGO workers to leave the profession in search of a job with better conditions that improves the quality of their personal life, including their wellbeing [73]. The retention of employees has been related to the degree of discretion that the workers of these organizations can develop, and with the number of administrative functions they should perform [74].

From the social perspective, Lam and Kuippers (2019) showed that the lack of clear organizational structures negatively affects the performance of the organization [75]. Some organizational factors which influence burnout are limited resources and low support [76,77]. In agreement with Wagner (2020), the informal social structure of NGOs should coincide with the formal one, or organizational performance will decline [78]. Regarding public supervision, Choi, Kim, Ullah, and Kang indicated that experience in the sector is essential in order to get good results [79]. A correct level of supervision allows workers to be conscious of what they contribute to the company [80,81].

## 3. Research Hypotheses

**Figure 1:**
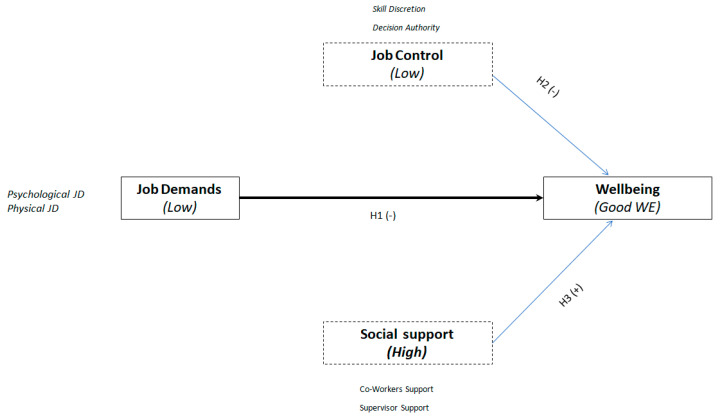
Research model. Source: prepared by the authors.

The three variables, job strain, and social support should interact with the wellbeing of NGO workers. In agreement with the bibliography analyzed previously, three research hypotheses are presented in Figure 1:

**Hypothesis 1** **(H1).** *As the job becomes more demanding, the wellbeing of NGO workers decreases*.

**Hypothesis 2** **(H2).** *As NGO workers have a higher degree of job control, their wellbeing increases*.

**Hypothesis 3** **(H3).** *As NGO workers have a higher degree of social support, their wellbeing increases*.

## 4. Methodology

### 4.1. Data Collection and Sampling

The European Working Conditions Survey (EWCS) has been carried out every five years since 1990, oriented to provide an overview of the workplace in the region. An interview based on a questionnaire is used in order to evaluate and quantify working conditions and to analyze relationships between their different aspects (the complete questionnaire can be found in the EFIW web 2019. https://www.eurofound.europa.eu/sites/default/files/page/field_ef_documents/6th_ewcs_2015_final_source_master_questionnaire.pdf). In the 6th survey, a multistage stratified sample was collected in all EU countries, five candidate countries, and two from Norway and Switzerland, using face-to-face interviews. EWCS has been widely used to explore working conditions in Europe due to its multidimensional nature [82,83]. From this database, a sample of NGO workers was selected, and after shelving the cases with missing data, 1000 records were finally used (the sample was selected using Q4—identifying NGOs). A brief description of the data is summarized with the following results: only 8.1% are self-employed, 33% are male, most of them are aged between 23 and 65 years, with a median of 45 years, similar for both sexes. Nearly 75% of the employees in the sample had permanent job contracts, and 61% of employees worked full-time, while the remaining 39% collaborated as a part-time activity. With NGOs, 25% of workers had a contract of limited duration, 1% had indefinite contracts, 5% were provided by a temporal work agency, just 1% were at an apprenticeship stage, and the rest were without any contractual relationship.

### 4.2. Measures

In the EWCS database, 34 items related to the proposed model were selected to analyze the causal factors of wellbeing in the workplace, plus six questions about personal conditions like gender, age, type of labor relation, and salary conditions. These 34 variables were classified into four groups related to four constructs or latent variables based on the structural equation model proposed in order to explain the causal factors of worker wellbeing (see Table 1). All measures were based on questions taken from the 5th EWCS. Even though some questions might not be identical to some used in other purpose-designed instruments, as in [49,84,85], the items in this survey were tested and proved to have good face validity, and many were adapted from validated instruments [86].

The latent variables introduced are three exogenous variables and one endogenous variable; these constructs and their links to the observable variables are listed in Table 1. The exogenous latent variables are

-Job demand (JD), which is related to 15 observable variables represented by JD1 to JD15. This construct, JD, includes physical (variables JD1 to JD9) and psychological aspects (variables JD10 to JD15) in the workplace. The job content questionnaire (JCQ), which collects questions related to the physical demands of the work environment, that is to say, what forms the natural habitat of the position, which, of course, may cause job risks of a physical and psychological nature. A total of thirteen questions were used to measure the risk of physical demands at work. High levels of job demand mean high levels of physical and physiological risk due to work conditions.

-Job control (JC) is linked to six observable variables named JC1 to JC6. This construct analyzes the intrinsic motivational ability of the position in terms of the demand of capabilities of a greater nature or, where appropriate, basic demands according to the tasks (monotonous or complex) that are developed in the position. Some of these questions are related to the work’s methods, the productivity required, and the possibility of choosing the type of work and applying their own ideas to learn new things. Three binary variables (Q53) were added to form a new variable related to the application qualities of the job (JC1), and three binary variables (Q54) related to the authority decisions were added to generate JC2; the rest are Likert five-point scale variables.

-Social support (SS) is related to seven variables from the EWCS questionnaire, from the assessment of aspects related to independence and the ability to make decisions, which the worker perceives in aspects such as the choice of tasks, functions, work methods, with questions such as “You can choose or change your work methods”, to the support of work colleagues. The support of supervisors analyzes the perception of the worker in relation to their immediate boss in aspects such as recognition, respect, help, or feedback of the work results using seven elements.

The endogenous variable of the structural equation models is a construct related to wellbeing (WB) in the workplace, as shown in Table 2. Six questions about the worker’s feelings are related to this latent variable (Table 2). It was created on the basis of the wellbeing index created by the regional office of the World Health Organization in Europe (WHO). According to [87], these are the five items of the index (WHO-5) that assess subjective psychological wellbeing.

To measure the internal consistency of the test items, Cronbach’s alpha was obtained for each group of variables and is summarized in Table 3.

### 4.3. Structural Equation Models

Structural equation models (SEMs) [88] are an extension of general linear models. It includes multiequational specifications to investigate interrelations and causal influences between different types of variables: latent or constructs and observed variables. The relations between the variables are represented in a graph, where there is usually a set of relationships between the latent variables, forming the structural model, and several factor-analysis-type models linking the observable variables with each construct. The correlation or covariance matrix of the observable variables is decomposed in the part explained by the full SEM and a residual matrix. The latent variables can be interrelated in noncausal ways or with oriented vectors representing causal influences. Additionally, the latent variables can act as exogenous in the measurable factor analysis models. Every endogenous variable, latent or observable, is completed by random disturbance terms that could be interrelated between them, originating additional parameters to be estimated. As in any multiequational econometric model, SEMs can arouse identification problems that originate from the noninterpretability of some relationships. This is solved by using some restrictions upon the set of parameters, restrictions that should be compatible with a global fit of the model. Some specialized software, like AMOS from IBM corporation (Mahwah, NJ, USA), perform identification checks, while several alternatives based on partial least square procedures avoid these tests, with the danger of obtaining without being aware of unidentifiable relationships. Once a model has been estimated, at least two types of validation diagnostics are necessary [88]. First, a global fit statistical test based on the asymptotic likelihood ratio statistic *G*^2^ (in AMOS terminology, CMIN), whose sampling distributions tend to a chi-square with the model degrees of freedom and a test over each estimated parameter (which reminds us of the *t*-test in regression analysis). Secondly, some goodness of fit measures ought to be calculated. These are categorized into several groups, and AMOS obtained most of the usual measures and an interval useful to judge the degree of fit. One extreme corresponded to the saturated model (a model with perfect fit and no degrees of freedom) and the other to the independence model (corresponding to a state of affairs of no relationships between the observable variables). This is useful to produce a clear meaning of statistics that do not have a bounded range. In the first group of goodness-of-fit statistics, the root mean-square error (RMSEA) is based on the residuals of the model, and it should be as close to zero as possible; the root mean-square of the normalized residuals can also be used. The second group of goodness-of-fit statistics compares the proposed (default) model to the independence situation. The Tucker–Lewis (NFI) and Bentler (CFI) indices should be as near as possible to one, which reminds us of the classical determination of the coefficient, and they are related to the proportion of covariance explained by the model. The third group depends on the likelihood function and includes the Akaike (AIC) and Schwarz (BIC) information criteria; the lowest possible values are associated with a better fit, and in this case, the lower limit calculated by the program is useful in interpreting their values. In each group, AMOS obtained several additional measures of fit. The model proposed, as detailed in the next sections, passes all the statistical global and parameter tests, and show a very good goodness-of-fit with all the different measures.

## 5. Results

**Figure 2:**
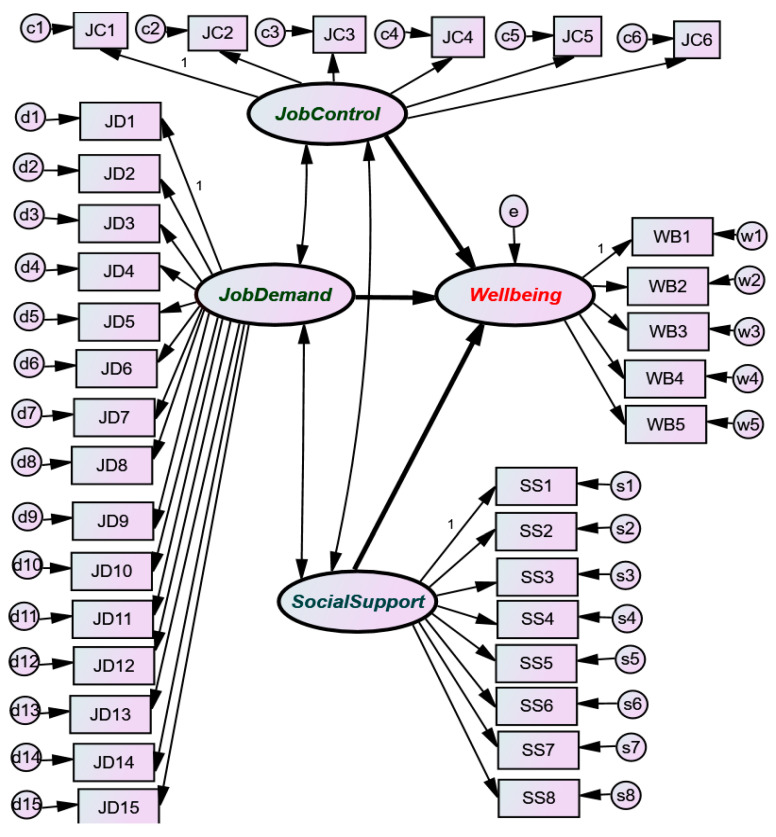
Structural Model.

A structural model is proposed in order to analyze the influence of wellbeing at work with different unforeseen variables such as controlling the work position, the demand for qualification, and the support or social recognition for this type of job (Figure 2). In the process of specification, various alternatives have been suggested in order the select the most congruent data. In the case of breaking down the nature of job control or decision authority into two constructs, identification problems arise in the structural model, so the simplified form is proposed by reducing the constructs to those included in the model.

Various alternative models have been estimated to reach the final specification, which has been kept as simple as possible, to achieve robustness. There are no identification problems in the final model.

Every latent variable is related to a set of observed variables, including all the variables linked to each construct, as specified in Table 1 and Table 2. The models, including the measurement variables, are, actually, factor analysis models. The exogenous variables of Job Control, Job Demand, and Social Support are interdependent but not causally related. They show causal influences upon the endogenous wellbeing construct. The random disturbances of each group of variables begin with the same letter: c, d, s, w, followed by a digit, and the random disturbances of Wellbeing are represented by e. They are supposed to be compatible with the usual a priori hypothesis admitted in structural modeling. As the sample size is quite large, the maximum-likelihood estimators involved are considered appropriate.

The AMOS software, linked to the SPSS 26 statistical package (Mahwah, NJ, USA) has been used to estimate the structural equation model (SEM), with input from the correlation matrix between the observable variables. The overall fit is tested using the likelihood ratio statistic, *G*^2^ = 344.26 (CMIN in AMOS terminology). Its *p*-value is 0.082, higher than the significance threshold α = 0.05, so the model can be globally accepted. The degrees of freedom are quite high (309).

The model parameters are tested with the usual critical-ratio (C.R.) statistics, obtained by dividing the coefficients estimates by their standard errors (S.E.). In the following table, the first three parameters, which are used to confirm our research hypothesis, correspond to the latent variable model. Then, the estimated coefficients of the factor analysis model complete the SEM. In the following Table 4, the coefficients of the estimated model are presented, and they are all significant, most of them with a limit probability of *p* < 0.001 (represented by ***) and the rest quite low, so they are kept in the model. As can be deduced from the model, job control and social support positively affect workers’ wellbeing, while job demands tend to lower this construct. In the factor analysis models linking the latent and observable variables, the estimated coefficients also present low *p*-values.

The latent Job Control, Job Demands, and Social Support variables are intertwined, as supported by the tests on their covariances, which are significant with *p*-values < 0.001, as stated in Table 5. These relationships could be the cause of the interaction effects on wellbeing.

Goodness-of-fit measures are presented for the proposed model (in AMOS terminology, the “default model”), the saturated model (the model with the perfect fit), and the independence model (the other extreme, representing the case of independence between all the observed variables). As will be seen, the goodness-of-fit measures for the proposed model are fairly close to optimum, corresponding to the saturated model and confirming the acceptability of the proposed model. The ratio CMIN/DF = 324.06/309 is considered acceptable. Measures based on population discrepancy are also indicative of the model fit; the root mean square error of approximation, RMSEA = 0.011, is close to zero, the optimum value, and below the usual maximum acceptable, 0.05. The comparison measures to a baseline model such as the normed fit index (NFI), the relative fit index (RFI), the incremental fit index (IFI), the Tucker–Lewis coefficient (TLI), or the comparative fit index (CFI) take (in all cases) values near the optimum (1).

The goodness-of-fit measures based on the information likelihood function are in line with previous results, accepting a good fit for the proposed default model; the Akaike information criterion (AIC), the Browne–Cudeck criterion (BCC), the Bayes information criterion (BIC), and the consistent AIC (CAIC) are close to their optimum values. The Akaike statistic is AIC = 916.261, which is close to the optimal value obtained with the saturated model; additionally, BCC = 937.028, which lies near the value associated with the saturated model.

In summary, all tests and goodness-of-fit measures tend to support the proposed model (Table 6).

## 6. Discussion

The main aim of this study is to use the JDCS model in an unusual working environment, that is, in NGO entities, which seem to have been neglected by human resource academic researchers, who may not be taking into account their importance and contributions to social problems. NGOs present a singular picture as their projects are nonprofit and oriented to create positive changes in society and communities on a global scale. For these institutions, the concept of developing the human being and the value of social change and development are essential to promoting a sustainable environment so that people can enjoy their lives [89]. The key to understanding why NGOs are so essential is the fact that, due to global shortcomings, there has been an increase in social problems, climate change, and population migrations, and these challenges require supranational responses [88].

The studies on wellbeing in the service sector are somewhat scarce [90]. This may be because, as indicated by Aguiar, Conde, and Diego (2018), it deals with a varied, complex, and dynamic sector, with a great ability to adapt to new social and economic realities [91]. Nongovernmental organizations are unique due to the fact that they are nonprofit and that the society needs them, a matter which is reflected in the workers of the sector, who present low job demands, low control, and high social support. The importance and social contributions of these entities and their employees are in clear contrast with the low demand for their aptitudes and results, and this undoubtedly affects motivation in the workplace and the low desire to continue in this kind of job.

Some studies, such as Ratuva, Compel, and Aguilar (2019), have indicated that, often, when a job is subject to inspection and under the state, it is institutionalized, and it is a job that can become routine, centralized, and slow, with limited resources [92]. Studies, such as that of Rogers, Amstrong, Sellars, and Manson (2008), have shown that the context in which the services sector operates is a “complex ensemble of cultural systems and institutional interactions” [93]. They share a basic premise of an increase in the degree of employee participation. These workers need to identify with the principles of empowerment, self-determination, social justice, human rights, inclusion, and equality in order to complete their job activities [94]. The International Federation of Social Workers (IFSW) shows that it is important that the workers in the NGO sector should act in multiple and innovative ways in their daily activity. NGOs survive within an unfair job system because their professionals are completely involved in internal factors as the defense of human rights, social action, and assessment of their results [95]. Therefore, these organizational structures are not oriented to produce a special level of wellbeing in their employees. A downgrade in this situation should affect employee motivation and the quality in the workplace, originating a desire to change jobs and affecting, thus, in the long-term, society as a whole.

This paper aims to show the importance of the analysis of wellbeing in NGO employees in the European context and relate several working conditions such as labor demands, control in the workplace, and the social support perceived. The three research hypotheses proposed are confirmed using a structural equation model. The worker’s control of his activities in the workplace is the main factor explaining the wellbeing perceived: an increase in this control produces a fall in wellbeing sensations. This is in accordance with previous results referred to in the literature, in line with a loss of motivation, burnout syndrome, and willingness to leave the present job. The results achieved show that NGO employees perceive an increase in the wellbeing sensation when they receive direct support from the organization. Additionally, the possibility of greater autonomy and control of their activities produces a direct positive effect on their wellbeing, but this situation is not frequent due to the usual centralized structure of these kinds of institutions, which, in practice, allow only limited room for individual initiatives.

The model supports the first hypothesis, specifically that the marginal effect produced by an increase in job demands (physical and psychological) decreases the wellbeing of employees in this sector by −0.387. An explanation of this result is determined by the fact that physical demands are a priority in the welfare system, due to risks related to people’s integrity in this profession [96]. Within the physical demands, the aspects that most influence the decrease of wellbeing are the physical risks that are properly stated (noise, vibration, temperature) and also chemical and biological risks. Specifically, the physical demands that most influence wellbeing are the noise that the worker suffers in the workplace (1.523), followed by high temperatures (1.432). For their part, psychological aspects such as working fast (1.126) and meeting deadlines (0.691) stand out among the most influential emotional demands. It would seem that to increase workers’ wellbeing, job demands should be promoted, as a higher motivation would result from incentives in doing new activities, but, at the same time, these initiatives could originate negative effects on the employee’s health because of an increase in work stress [97]. According to Deloffre (2019), the social visibility of NGOs can be found with an increase in the demand for new activities, as this shows the commitment and impact of these institutions towards society [98]. From this perspective, public trust is a consequence of the tasks accomplished by NGOs that are related to social problems not solved by governments. This is often bourne in a harsh political climate and complemented with very limited financial rewards [99]. In consequence, this objective of an increase in labor demands could have a limited effect due to the increase in the maintenance costs of their human resource structure.

Hypothesis 2 is tested within the structural model; the negative causal relationship that exists between Job Control (0.287) and Wellbeing can be confirmed by the results obtained with the test upon its coefficient. Specifically, the results of this study suggest that in order to increase the wellbeing of NGO workers, they have to present high levels of ability in decision-making (1.119) and independence (0.840). NGOs are hierarchically integrated organizations; even in the cases of the federations of several groups in different countries, there is a transfer of authority to a central decision-making structure for global affairs. These types of agreements tend to evolve towards a central body. The reasons to explain this trend are linked to the political aspects within the organizations, which tend to adapt to the particular interests of the stakeholders involved, diverging from the basic aspect of providing services to society [100]. In accordance with Meyer, Zhang, and Ding (2018), the studies regarding the negotiation between the international NGOs and the Chinese government emphasize the use of personal connections [101]. This article specifically states that international NGOs should be free of external control through the creation of institutional links with the government, which helps NGOs reduce their dependency, allowing for the improvement of their workers’ freedom of action.

Finally, the third hypothesis is confirmed by the test upon the corresponding coefficient in SEM. It could be further confirmed by observing the aspects related to social support (supervisor and coworkers), which are important for workers and positively influence their wellbeing (0.088), although in a lesser measure than the previous hypothesis. From the perspective of the supervisor, the aspects which most involve the workers’ wellbeing are the feedback they may provide (1.620) and the support for professional development (1.5). However, recognition from colleagues has less influence than that of a supervisor (0.334). A similar conclusion was reached by Seponski and Jordan (2018), which showed the difference between the supervisors of international NGOs in Cambodia and foreigners, due to cultural reasons [102]. A supervisor should help the workers being supervised to organize their ethical values and dilemmas [103,104].

As usual in social science empirical research, the results obtained in this study should be interpreted with caution. First of all, the methodology used is based on the JDCS model. For its implementation, EWCS was used, which is constructed on self-assessed measures and, as such, susceptible to bias. However, [103] indicated that even though surveys based on self-assessed measures may be used, it supports the strain hypothesis of the model to a greater degree and consistency than other articles that use more impartial assessment tools. Secondly, the research has a specific sociogeographic limit (Europe), with a very contrasting job environment and labor regulations compared to other countries, which conditions the results and the basic variables of the JDCS model. On this basis, it would be inappropriate to generalize the results to other job environments outside developed nations. A future line of research should analyze different job categories in the industry of the NGO sector (for example, according to categories) and analyze the influence of different geographical areas, their comparison, and the influence of the cultural environment. Finally, workers that are not from Europe, but work with European NGOs, may have a different organizational or management system compared with the framework in this study, and this would be a difficulty in the task of providing general guidelines to the management of human resources.

## 7. Conclusions

An employee in NGOs is usually someone with a characteristic profile that is different from the usual worker in an industrial or service company. In accordance with Bidee, Vantilbourg, Pepermans, Huybrechts, Willems and Jeger, and Hofmands (2013), NGO employees are linked to the nonprofit character of these institutions, and they generally feel that their activities are agreeable and interesting [104]. They consider this sector an added-value in addition to an ordinary labor market job. To analyze a human being’s intrinsic or internal motivations, it is necessary to find the links to their psychological needs, such as autonomy, competence, and social relations; these needs are to be fulfilled with an engaging activity [104]. In fact, under this functional approach developed by Clary (1992) [105], and continued by Lorente-Ayala (2019), workers in this sector do not seek a priori self-directed personal benefits [106,107], but they aim to cover their psychological needs over other working conditions. Some workers have a high motivation to help society; they can search for a high degree of wellbeing, mainly due to internal and personal factors, despite the working conditions established by the NGO (Wearing and McGehee, 2013) [108]. Academic literature shows that both motivations are compatible [109,110,111]. Internal factors are highly important for NGO employees [106,107]. In fact, several publications have pointed to a certain degree of narcissism associated with this work environment [112,113], as it is linked to personal motivations and sensations of transcendence in their activities.

It is typical of this sector of workplace situations, where internal values overcome external factors, causing a smoothing in the impact inferred upon worker wellbeing. Several papers in scientific journals have supported the importance of these internal values in NGO employees [108], and, in contrast to this situation, they have also pointed to a high level of turnover in the participation of their workers and volunteers [114]. An explanation of these circumstances can be associated with the diminishing importance of the internal factors, with decreasing importance in the workplace [115]. In addition to these trends, the composition of the workforce in NGOs does not provide long-term wellbeing, motivations, and work-engagement for their employees. In fact, these characteristics can even disappear or diminish to a point where stress and loss of motivation are the natural consequences, originating further abandonment and turnover in the workforce.

This study produces practical and theoretical implications for NGOs in Europe. At a theoretical level, it shows how the JDCS model works in an NGO institution, where a high degree of commitment is required on behalf of the organization, a matter which may contribute decisively to improving the wellbeing of the professionals of this sector, which, in fact, is associated to the working conditions in this kind of institution (low demand, low control, and recognized or supported). The lack of developments around this research niche may be due to the fact that workers in this sector prioritize doing their work against scientific analysis or the fear of souring the image of NGOs. As a consequence, the literature on NGOs is limited in this area due to the reduction of funds that implies the loss of jobs. From a practical perspective, the results of this article confirm the concept that job demands are not the only variables that affect wellbeing, especially when the workers perceive job control and/or social support of the organizations. As a result, the sector should care about factors from the workplace that affect wellbeing.

NGOs tend to prioritize a work culture in which workers are mainly concerned with the specific actions they translate to society. In modern times, work-environments value efficiency and compactivity in their activities. NGOs are based on the belief that the strength of their organizations relies on the internal factors of their human resources. However, this paradigm is not sufficient for these institutions as their laudable intentions and motivations, as an intrinsic factor, are starting to look out-of-date to achieve their aims efficiently.

In fact, most NGOs start as small informal structures, where the human resource structure is approached informally, a factor that later collides with organizational growth, causing a decrease in wellbeing. Furthermore, when an NGO is successful and focuses on organizational expansion, the investment that occurs in human resource systems is the result of unplanned growth because it is determined by an unexpected strategic change. Modern NGOs must adapt their human resource strategies to increase the employee’s work-engagement, productivity, and efficiency. External factors have also to be taken into account to avoid management errors and to increase worker protection to burnout, absenteeism, lack of motivation, and low commitment.

Following this idea, people who work in this sector look for alternatives of action to those established by the conventional or private system. Thus, regulation by multinational institutions is necessary or, at least, convenient in areas such as hiring and salary policies, oriented to increase employee protection and professionalism. Psychological factors are not considered as fundamental in hiring policies.

In their ordinary management, it is necessary to include new long-term strategies, with periodical assessments, including the social issue priorities of NGOs. Job control is primarily linked to public and private sector funding and to the ordinary protocols in daily actions. The audit of financial flows and the expected transparency have to be linked to project objectives and the achieved objectives and results. Control protocols should be flexible, with deadlines oriented to increase worker autonomy. Hiring policies should be oriented to the activities developed, avoiding nonplanned growth, and aiming to retain qualified workers. Some transnational organizations could be of help by proposing regulations and audit procedures and the assessment of the needs of the workforce and its qualifications.

These organizations are under pressure so that the funds destined for hiring are meant to revert to the people in need or to the geographical area where they operate, causing a fall in worker wellbeing by lowering the suitability and professionalism of the candidates. Human resource managers of these entities should explore these topics to be able to plan some strategic design regarding new jobs and management tools. It implies direct contact with the users, and the assessment of the quality of the goods provided. In addition, the directors of the NGOs should look for and develop a work environment based on cooperation and an organizational culture of support; they should encourage teamwork. The investment in the improvement and ability of teams influences features such as organizational climate and social support, which produce advances within the organization and increase the approach towards assistance.

## Figures and Tables

**Table 1:** European Working Conditions Survey (EWCS) variables linked to exogenous constructs.

Construct	Concept	Questions Codes and Text	
Job Demands (JD)	Physical Job Demands	JD1	Vibrations produced by hand tools, machines
		JD2	Noises so loud that you have to raise your voice to speak to people
		JD3	High temperatures that make you sweat even when you are not working
		JD4	Low temperatures, either inside the building or outside
		JD5	Breathing fumes or gases, dust, ….
		JD6	Breathing vapors, such as solvents or thinners
		JD7	Manipulating or having the skin in contact with chemical products or substances
		JD8	Other people’s tobacco smoke
		JD9	Handling or having direct contact with materials that may be infectious
	Psychological Job Demands	JD10	Working at very high speed
		JD11	Working to tight deadlines
		JD12	You have enough time to get the job done
		JD13	Situations emotionally disturbing
		JD14	You Know what is expected of you at work
		JD15	Your job requires that you hide your feelings
Job Control (JC)	Application Qualities	JC1	Solving unforeseen problems on your own
			Monotonous tasks
			Learning new things
	Authority Decision	JC2	Your order of tasks
			Your methods of work
			Your speed or rate of work
		JC3	You are consulted before objectives are set for your work
		JC4	You have a say in the choice of your work colleagues
		JC5	You are able to apply your own ideas to your work
		JC6	You can influence decisions that are important for your work
Social Support (SS)	Authority Support (by your boss)	SS1	Respect you as a person
		SS2	Give you praise and recognition when you do a good job
		SS3	Is successful in getting people to work together
		SS4	Is helpful in getting the job done
		SS5	Provide useful feedback on your work
		SS6	Encourage and supports your development
		SS7	Manager’s help and supports
	Coworkers Support	SS8	Help and support

**Table 2:** EWCS variables linked to the wellbeing construct.

Construct	Concept	Questions Codes and Text	
Wellbeing (WB)	Feelings at the workplace	WB1	Good spirits and cheerful
		WB2	Calm and relaxed
		WB3	Active and vigorous
		WB4	Fresh and rested
		WB5	Interesting activities

**Table 3:** Internal consistency.

Construct	Variables	Cronbach’s α
Job Demands	JD1 to JD14	0.725
Job Control	JC1 to JC6	0.679
Social Support	SS1 to SS8	0.899
Wellbeing	WB1 to WB5	0.855

**Table 4:** Structural equation model (SEM) coefficient estimates and tests.

Dependent Variables		Causal Variables	Estimate	S.E.	C.R.	*p*
Wellbeing	←	Job Control	0.208	0.043	4.841	***
Wellbeing	←	Job Demand	−0.387	0.071	−5.422	***
Wellbeing	←	Social Support	0.088	0.039	2.277	0.023
JD1	←	Job Demand	1.000			
JD2	←	Job Demand	1.523	0.108	14.144	***
JD3	←	Job Demand	1.432	0.109	13.184	***
JD4	←	Job Demand	1.076	0.096	11.203	***
JD5	←	Job Demand	1.188	0.080	14.921	***
JD6	←	Job Demand	0.937	0.069	13.488	***
JD7	←	Job Demand	1.234	0.100	12.346	***
JD8	←	Job Demand	1.314	0.094	13.939	***
JD9	←	Job Demand	1.030	0.137	7.524	***
JD10	←	Job Demand	1.126	0.145	7.761	***
JD11	←	Job Demand	0.691	0.150	4.617	***
JD12	←	Job Demand	−0.554	0.096	−5.751	***
JD13	←	Job Demand	0.694	0.125	5.569	***
JD14	←	Job Demand	−0.116	0.057	−2.013	0.044
JD15	←	Job Demand	0.215	0.107	2.005	0.045
JC1	←	Job Control	1.000			
JC2	←	Job Control	0.058	0.029	2.001	0.046
JC3	←	Job Control	0.544	0.049	10.992	***
JC4	←	Job Control	0.599	0.147	4.078	***
JC5	←	Job Control	0.840	0.064	13.041	***
JC6	←	Job Control	1.119	0.081	13.783	***
SS1	←	Social Support	1.000			
SS2	←	Social Support	1.391	0.054	25.939	***
SS3	←	Social Support	1.362	0.063	21.589	***
SS4	←	Social Support	1.482	0.072	20.611	***
SS5	←	Social Support	1.620	0.068	23.878	***
SS6	←	Social Support	1.508	0.062	24.306	***
SS7	←	Social Support	0.638	0.057	11.142	***
SS8	←	Social Support	0.334	0.067	4.979	***
WB1	←	Wellbeing	1.000			
WB2	←	Wellbeing	1.093	0.052	20.894	***
WB3	←	Wellbeing	1.192	0.054	22.115	***
WB4	←	Wellbeing	1.363	0.065	20.850	***
WB5	←	Wellbeing	1.054	0.064	16.495	***

***: *p* < 0.001.

**Table 5:** Covariances between the exogenous constructs.

COVARIANCES			Estimate	S.E.	C.R.	*p*
Job Control	⇄	Job Demand	−0.124	0.020	−6.104	***
Job Demand	⇄	Social Support	−0.076	0.016	−4.819	***
Job Control	⇄	Social Support	0.270	0.033	8.066	***

***: *p* < 0.001.

**Table 6:** Information on the theoretic measures of goodness-of-fit.

Model	AIC	BCC	BIC	CAIC
Default model	916.261	937.028	2319.879	2605.879
Saturated model	1190.000	1233.205	4110.114	4705.114
Independence model	13,008.093	13,010.562	13,174.957	13,208.957
